# Synergy of AERONET and MODIS AOD products in the estimation of PM_2.5_ concentrations in Beijing

**DOI:** 10.1038/s41598-018-28535-2

**Published:** 2018-07-05

**Authors:** Disong Fu, Xiangao Xia, Jun Wang, Xiaoling Zhang, Xiaojing Li, Jianzhong Liu

**Affiliations:** 1 LAGEO, Institute of Atmospheric Physics, Chinese Academy of Sciences, Beijing, 100029 China; 2 College of Earth Sciences, University of Chinese Academy of Sciences, Beijing, 100049 China; 3 Collaborative Innovation Center on Forecast and Evaluation of Meteorological Disasters, Nanjing University of Information Science & Technology, Nanjing, 210044 China; 4 Department of Chemical and Biochemical Engineering, Center for Global and Regional Environmental Studies, and Informatics Initiative, The University of Iowa, Iowa City, IA 52241 USA; 5 School of Atmospheric Sciences, Plateau Atmosphere and Environment Key Laboratory of Sichuan Province, Chengdu University of Information Technology, Chengdu, 610225 China; 6 National Satellite Meteorological Center, China Meteorological Administration, Beijing, 100082 China; 7 Beijing Meteorological Bureau, China Meteorological Administration, Beijing, 100081 China

## Abstract

Satellite aerosol optical depth (AOD) is widely used to estimate particulate matter with aerodynamic diameter ≤2.5 µm (PM_2.5_) mass concentrations. Polar orbiting satellite retrieval 1–2 times each day is frequently affected by cloud, snow cover or misclassification of heavy pollution. Novel methods are therefore required to improve AOD sampling. Sunphotometer provides much more AODs than satellite at a fixed point. Furthermore, much of the aerosol pollution is regional. Both factors indicate that sunphotometer has great potential for PM_2.5_ concentration estimation. The spatial representativeness of the Aerosol Robotic Network (AERONET) AOD at Beijing site is investigated by linear regression analysis of 13-year daily paired AODs at each grid from Moderate Resolution Imaging Spectroradiometer (MODIS) on Aqua and Beijing AERONET. The result suggests a good correlation for the whole Beijing Administrative region, with regional mean correlation coefficient exceeding 0.73. Pixel AODs are then estimated from AERONET AOD using linear equations, which are verified to have the same accuracy as that of MODIS AOD. Either AOD from MODIS retrieval or estimation from AERONET AOD in the absence of MODIS pixel AOD is finally used to predict PM_2.5_ concentration. Daily AOD sampling in average is enhanced by 59% in winter when MODIS AODs are very limited. More importantly, synergy of AERONET and MODIS AOD is able to improve the estimation of regional mean PM_2.5_ concentrations, which indicates this method would play a significant role in monitoring regional aerosol pollution.

## Introduction

Application of satellite-derived aerosol optical depth (AOD) to estimate ground-level particulate matter with aerodynamic diameter ≤ 2.5 µm (PM_2.5_) has advanced dramatically since it was initiated more than one decade ago. In early work, Wang and Christopher^1^ demonstrated the potential of using satellite-based AOD from Moderate Resolution Imaging Spectroradiometer (MODIS) to derive PM_2.5_ concentrations. Further studies have attempted to improve the PM_2.5_-AOD relationship through many linear and nonlinear statistical models in which additional parameters such as meteorological and environmental parameters are introduced to develop multiple linear regression model^2^, geographically weighted regression model^3^, land use regression models^4^, artificial neural networks^5^, chemical transport models (CTM)^6^, mixed effects model^7^.

Although these studies differ to some extent in their methodologies, the fundamental requirement of these methods is the same, i.e., satellite AOD products should be available; otherwise, it is all but impossible to derive PM_2.5_ from AOD with sufficient observational constraint. While satellite-predicted PM_2.5_ provides larger spatial coverage than ground-based measurements, its availability, 1–2 times per day by polar orbiting satellites at most, is frequently affected by clouds, snow cover and even heavy aerosol pollution (misclassification)^7^. For example, there were only about 120 days with AOD-PM_2.5_ matchups a year for MODIS products in Beijing^8^. Gupta *et al*.^9^ found that satellite daily AODs were generally available less than 50% of the time over 38 locations in the southeastern United States. Hence, solving the under sampling problem is one of fundamental requirements for the improvement of PM_2.5_ estimation from space^10^. Several methods have been made to solve this issue. Kloog *et al*.^11^ estimated daily PM_2.5_ for grid cell without AOD data by using the mean PM_2.5_ levels from nearby grid cells. A combined MODIS and MISR AOD has been used to improve AOD sampling^3^. Lu *et al*. proposed a method to estimate missing AODs by assuming a linear relationship between PM_2.5_ and AOD^12^. The methods mentioned above require either surface PM_2.5_ measurements to constrain the AOD filling or more satellite products.

Different from space-borne sensors, Aerosol Robotic Network (AERONET) has been providing robust AOD measurements in good temporal resolution for nearly two decades. More importantly, the boundaries of the region resembling AOD temporal variability as that at AERONET sites vary between 200 and 500 km depending on their specific locations, which indicates temporal variation of AOD at AERONET sites could be representative for a larger region^13–15^. This deduction is reasonable since the temporal variation of AOD is overwhelming determined by weather conditions that are able to lead to a coherent variation of AOD in a fairly large area. For example, a stable stagnant condition favors for a regional haze, on the contrary, a cold front always disperses large-spread haze dramatically. Both phenomena are often observed in North China Plain (NCP). Therefore, it is not surprising that a high agreement between spring AOD at Beijing and Xianghe has been reported^16^. A close connection between spatial distribution of AOD and the circulation types has also been shown^15,17^. Changes of temporal and spatial emissions should have played a minor role in the AOD representation of one location relative to weather conditions. This opens opportunities to enhance PM_2.5_ estimation from AOD if we can establish a robust synergy between spatial converge from satellite and temporal coverage from AEROENT for estimating PM_2.5_.

**Figure 1:**
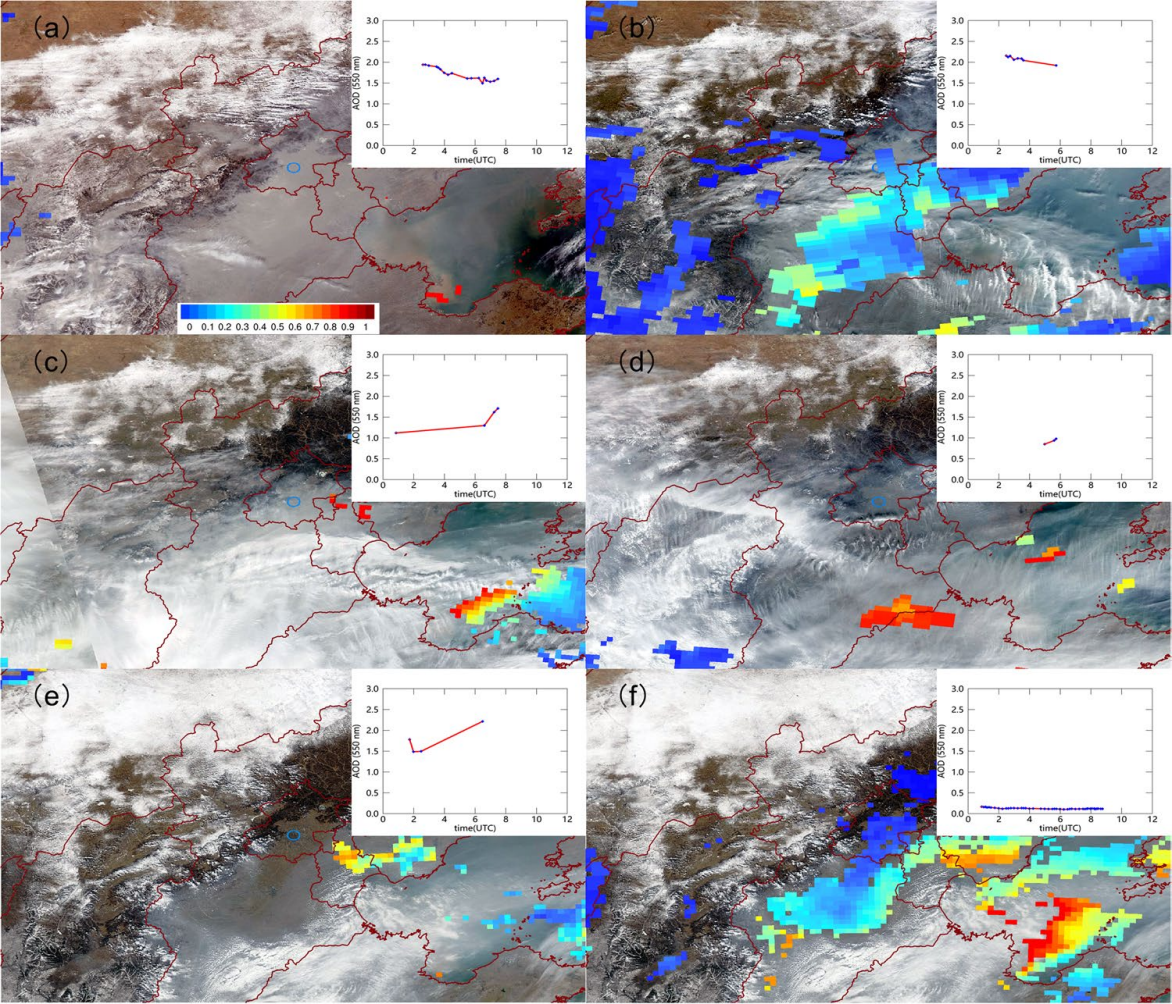
MODIS/Aqua RGB images in North China Plain overlaid with MODIS AODs as well instantaneous AERONET AODs at 550 nm on Feb. 14, 2014 (**a**); Feb. 15, 2014 (**b**); Feb. 16, 2014 (**c**); Feb. 17, 2014 (**d**); Feb. 18, 2014 (**e**) and Feb. 19, 2014 (**f**). The figure was generated in ArcMap10.2.

Beijing, the capital of the largest developing country in the world, China, has been suffering from heavy air pollution in recent years, especially in winter. A persistent regional air pollution episode occurred in winter of 2013 as recorded by a regional air quality monitoring network^18,19^, however, very few MODIS AOD retrievals are available for the estimation of a fine spatial variation of PM_2.5_. Heavy aerosol pollution is probably misclassified into clouds by the MODIS cloud discrimination algorithm since aerosol signal is to some extent close to that of clouds. Figure 1 presents examples of MODIS AOD retrievals under different conditions in NCP. Missing MODIS retrievals are likely due to clouds (on February 15) or misclassification of heavy haze to clouds (on February 14, 16, 18). On the contrary, AERONET AODs are available due to its high temporal resolution, especially on polluted days. Thus, AERONET AODs show their potential in the estimation of PM_2.5_ under these conditions.

Here we evaluate the representative spatial boundaries of AERONET-derived AODs at Beijing station (39.97°N, 116.38°E) and investigate its potential for the estimation of PM_2.5_ concentration at a regional scale. MODIS/Aqua daily level-2 AOD products in Beijing area from 2002 to 2014 are firstly interpolated into a regular gridded product with a spatial resolution of 0.1°. Linear equations have then been established from simultaneous daily AERONET AODs at Beijing and gridded MODIS AODs in the Beijing Administrative area, i.e., we create a distinct linear equation between Beijing AEORNET AODs and MODIS AODs at each grid. These equations are then used to fill missing MODIS AODs from AERONET AODs. Spatial distribution of PM_2.5_ concentration is finally estimated from a mixed effects model. Validation shows that this method is robust in the Beijing Administrative area that suggests a great potential of AERONET AOD products for monitoring PM_2.5_ concentration, especially in heavily polluted regions.

## Result

### AOD sampling enhancement by the synergy of AERONET and MODIS

**Figure 2:**
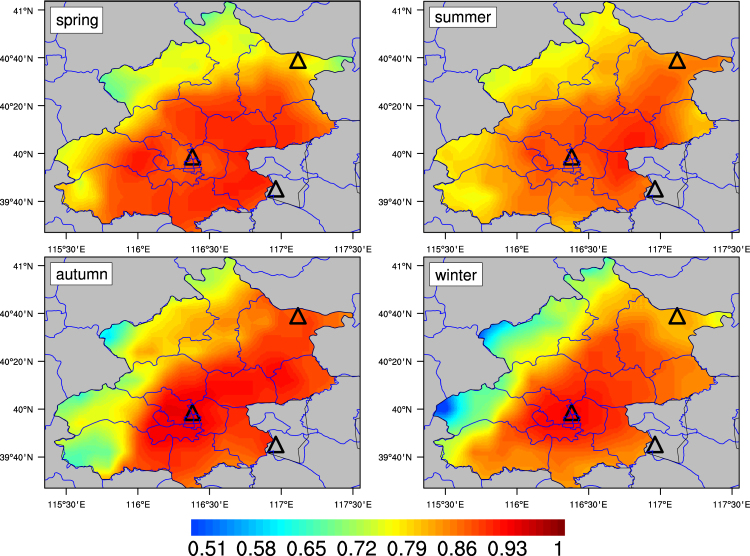
Seasonal correlation maps between daily-paired MODIS gridded AODs and AERONET AODs at Beijing for 2002–2014. The figure was produced using NCL. The map was created using ArcGIS 10.2 (ESRI Inc. Redlands, California, USA).

Figure 2 shows spatial distribution of correlation coefficients (R) between MODIS gridded AODs in the entire Beijing administrative area and Beijing AERONET AODs. Seasonal mean R values are 0.73 ± 0.14, 0.76 ± 0.09, 0.78 ± 0.11, 0.74 ± 0.14 for spring, summer, autumn and winter, respectively. As expected, R values decrease as a function of distance from the site. Meanwhile, R presents a decreasing gradient from eastern to western region (especially in winter), likely due to their differences in topography, land use and transport path. Spatial variation of paired data points should also contribute to this result.

**Figure 3:**
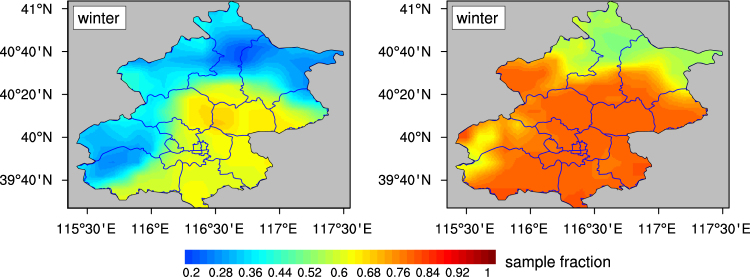
The ratio of days with AOD values to total day numbers before data fusion (**a**) and after data fusion (**b**) in the winter of 2013. This figure was processed using NCL. The map was created using ArcGIS 10.2 (ESRI Inc. Redlands, California, USA).

Figure 3 presents the performance of data fusion method during the winter of 2013. Before data fusion, MODIS retrieves AOD at approximately 50% of probability (regional mean). The retrieval percentage shows a spatial variation, ranging from about 20% in north to about 70% in south. After data fusion, regional mean AOD sampling over entire area increases to 81%. More specifically, AOD sampling substantially increases by ~50% in west and by ~40% in north.

**Figure 4:**
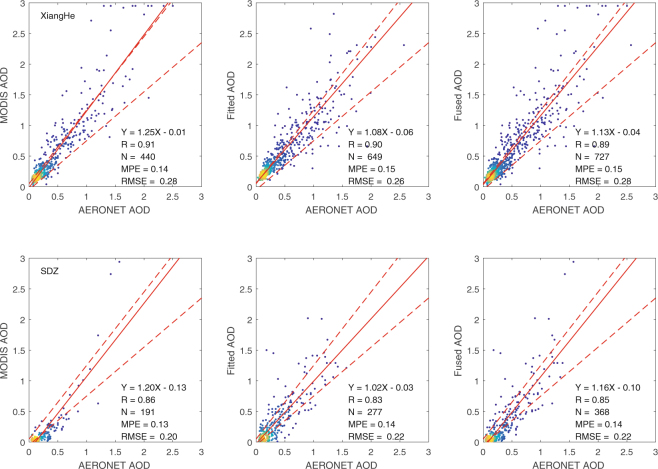
Comparison of MODIS only (left), linearly fitted (center) and fused AOD (right) to sunphotometer AOD data in the winter at Xianghe (upper) and SDZ (bottom) during 2005–2011. The solid lines represent the slopes of linear regression and the dotted lines the expected MODIS aerosol retrieval errors (±0.05 ± 0.20 × AOD). MPE and RMSE represent the absolute difference and root mean square error, respectively. This figure was produced using MATLAB.

MODIS and fused AODs were compared with independent sunphotometer measurements at Xianghe and SDZ (Fig. 4). MODIS AOD at the grid closest to the station is used to compare with sunphotometer daily-mean AOD. MODIS works very well in the retrieval of AOD at Xianghe and SDZ, with R of 0.91 (data points of 440) and 0.86 (data points of 191), respectively. 70.5% and 48.7% of MODIS AODs falling within the expected uncertainty of ±0.05 ± 20% × AOD at Xianghe and SDZ. MODIS AODs are closer to ground truth at Xianghe than at SDZ. This is likely associated with complex terrain at SDZ. The accuracy of AOD estimation from the synergy of AERONET and MODIS AOD is close to that of the MODIS retrieval. The mean prediction error (MPE) and root mean square error (RMSE) fused versus sunphotometer AOD are similar as those between MODIS and sunphotometer AOD. Fusion of AERONET-derived pixel AOD and MODIS AOD results in an increase of AOD sampling by 65% at Xianghe and 93% at SDZ in the winter (Fig. 4), which definitely would be expected to improve PM_2.5_ estimation from AOD.

### PM_2.5_ prediction

**Figure 5:**
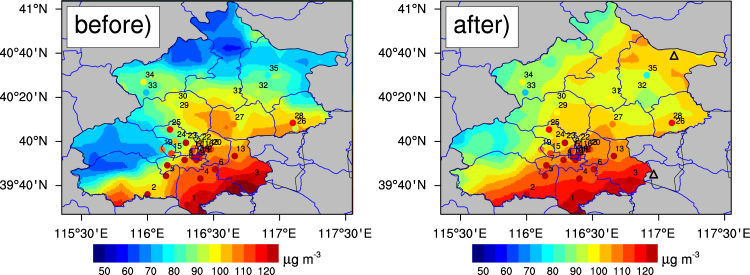
PM_2.5_ concentration maps retrieved using only MODIS AOD (left) and fused AOD data (right) in the winter of 2013. The corresponding mean concentrations at 35 sites are dotted on the figures. The figures were produced using NCL. The map was created using ArcGIS 10.2 (ESRI Inc. Redlands, California, USA).

The mean PM_2.5_ concentrations in the winter of 2013 were estimated using the satellite-based and fused AOD values (Fig. 5). Averaged satellite-derived PM_2.5_ concentrations over the entire area were 95.5 ± 67.8 μg m^−3^ and 104.3 ± 74.6 μg m^−3^ for these two datasets. PM_2.5_ values in southwest and north estimated from the fused AOD are larger than those from MODIS AOD by >20 μg m^−3^, which is mainly because AOD sampling increases substantially in these sub-regions by the fusion method. This result indicates that PM_2.5_ is probably underestimated if MODIS only AODs are used due to its under-sampling of AODs.

**Figure 6:**
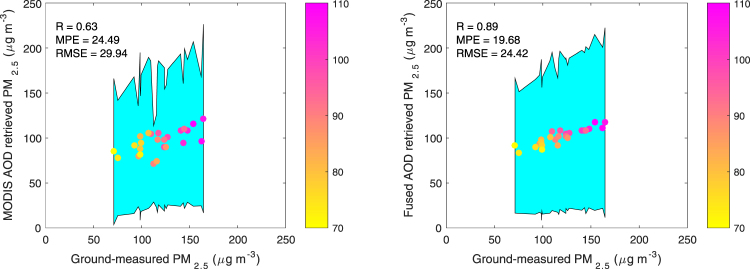
Scatter plots of the MODIS AOD derived PM_2.5_ (left) and the fused AOD derived PM_2.5_ (right) against ground-observed PM_2.5_ at 25 sites in the winter of 2013. The cyan shaded area represents the standard deviations of the retrieved PM_2.5_ and the color bar indicates the standard deviations of the ground-measured PM_2.5_. This figure was produced using MATLAB.

Improvement of PM_2.5_ estimation as a result of AOD sampling enhancement by fusion of AERONET AODs is clearly shown in Fig. 6, the scatter plot of station PM_2.5_ measurements and estimations at 25 stations. To evaluate the performance of both AOD datasets, we adopt the cross validation (CV) method. Here we collect collocate PM_2.5_ and AOD data at 25 stations. Only data at 24 stations are used to train the model while the data at the remaining station are used to evaluate the model each time. This leave-one-out process was repeated for each of the 25 sites, which follows the same procedure as previous study for cross-validation^10^. R between measured and MODIS AOD derived PM_2.5_ is 0.63. The MPE and RMSE are 24.5, 29.9 μg m^−3^, respectively. Much better performance of the fused AOD in the derivation of PM_2.5_ is evidenced by increased R (0.89), decreased MPE (19.7 μg m^−3^) and RMSE (24.4 μg m^−3^).

**Figure 7:**
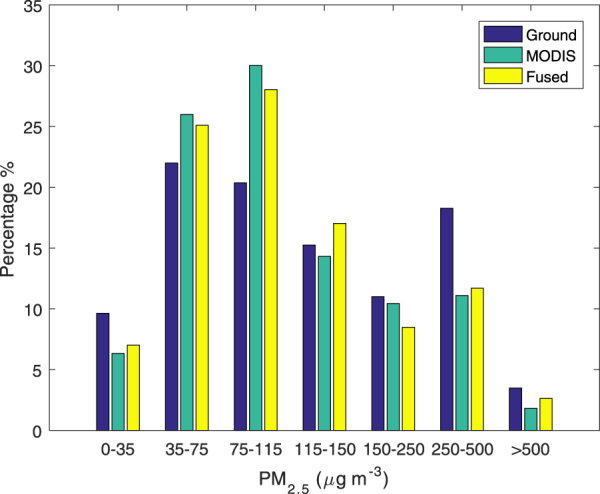
Histogram of the PM_2.5_ from three datasets, ground-level (left), MODIS AOD derived (center) and Fused AOD derived (right) PM_2.5_ concentrations. This figure was produced using MATLAB.

Figure 7 presents the histogram of three PM_2.5_ datasets at 25 stations, namely, ground measurement, estimations from fused and MODIS AOD. Compared with MODIS AOD derived PM_2.5_, Fused-AOD derived PM_2.5_ shows a histogram of PM_2.5_ much closer to that of ground measurements. The correlation coefficient and the mean absolute difference between ground measurements and fused-AOD derived PM_2.5_ are 0.90 and 3.59%, respectively, which are less than those between ground observations and estimations from MODIS AOD (0.88 and 3.90%).

The method is also applied in other three seasons. PM_2.5_ estimation has also been improved as a result of enhancement of AOD sampling by the AOD fusion, although the improvements are less than that in the winter. PM_2.5_ concentrations estimated from the fused AOD are all closer to ground measurements at 25 stations than those from MODIS AOD only (Table 1). Both mean PM_2.5_ and its standard deviation (temporal variability) estimated from fused AOD increase to some extent to approach to that of ground measurements.

**Table 1:** Statistics over 25 sites for retrieved results based on three datasets.

	PM_2.5_(G)	PM_2.5_(M)	PM_2.5_(F)
Spring	81.5 ± 59.5	79.7 ± 51.9	80.5 ± 53.0
Summer	67.2 ± 47.0	57.2 ± 35.9	63.3 ± 39.6
Autumn	99.1 ± 87.4	85.0 ± 63.0	87.7 ± 65.6
Winter	120.58 ± 90.66	95.5 ± 67.8	104.3 ± 74.6

(PM_2.5_(G), PM_2.5_(M) and PM_2.5_(F) refer to PM_2.5_ concentrations derived from Ground measurements, MODIS AODs and fused AODs, unit: μg m^−3^).

## Discussion and Conclusions

We use a statistical analysis to compare the AOD products from MODIS and AERONET Beijing between 2002 and 2014. The correlation analyses indicate that AOD at AERONET site can be used as representative of temporal variability for a larger region around its location. Grid AOD is then estimated from AERONET AOD at Beijing based on a linear regression analysis. The fused-AOD dataset provides a relatively higher temporal coverage in the winter (81%) instead of 50% days by MODIS only retrievals. PM_2.5_ concentration estimation using MODIS only AOD data resulted in an underestimation of PM_2.5_. PM_2.5_ concentrations calculated by the mixed effects model based on improved AOD sampling increased by 0.8, 6.1, 2.7, 6.5 μg m^−3^ in the spring, summer, autumn and winter, respectively.

The method in this study to fill missing MODIS AOD can supply more AOD data into chemistry models and model assimilations, provide good spatial and temporal coverage of PM_2.5_ concentrations based on increasing AOD-PM_2.5_ matchups, and offer better estimations of PM_2.5_ variability for epidemiological studies. Although only MODIS/Aqua data (13:30 local standard time) are used to generate the correlation map, using this map to calculate AOD at other times of the day may also be promising since the temporal variation of AOD is small. For example, Mishra *et al*.^13^ used the linear statistical model derived from MODIS/Aqua to prediction the spatial distribution of aerosol optical depth of MODIS/Terra. The result showed that the statistical model errors were generally below ~12%.

It should be noted that this method is highly dependent on the spatial representativeness of ground site and thereby optimal deployments of ground observations can enlarge the application of data fusion^14^. Besides, changes in spatial emissions over the domain in the past years may also play a role in the spatial correlation relationships that needs further study.

## Data and Methods

### PM_2.5_ Data

**Figure 8:**
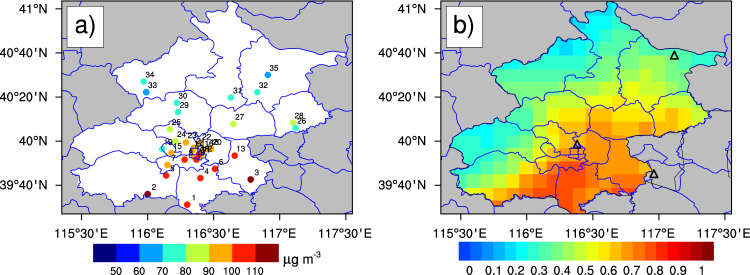
(**a**) Annual mean PM_2.5_ concentrations at 35 sites during Dec. 2013 to Nov. 2104; (**b**) Spatial distribution of the 10-km MODIS AOD in Beijing overlapped by 3 sunphotometer stations (black triangles) during Dec. 2013 to Nov. 2104. The figure was produced using NCL. The map was created using ArcGIS 10.2 (ESRI Inc. Redlands, California, USA).

Hourly PM_2.5_ concentrations from December 1^st^ 2013 to November 30^th^ 2014 at 35 sites are available online (http://zx.bjmemc.com.cn/) (Fig. 8) and daily-mean PM_2.5_ concentrations are calculated from hourly measurements within a day. Automated monitoring systems are installed at each site to measure ambient concentration of SO_2_, NO_2_, O_3_, CO, and PM_2.5_ and PM_10_ according to China Environmental Protection Standards. PM_2.5_ concentrations are measured by the Tapered Element Oscillating Microbalance method (TEOM). The TEOM’s filter is heated to avoid particle-bound water that may result in a slight underestimation of PM_2.5_ mass concentration owing to volatilization of semi-volatile material^20^. Inter-comparison of PM_2.5_ concentrations from the Beijing U.S. Embassy and the nearby Ministry of Environmental Protection site indicated that these two data sets were in good agreement in the temporal variation but the former was slightly higher than the latter since the beta attenuation monitor was used at the Beijing U.S. Embassy^21^.

### Modis AOD

Two Moderate Resolution Imaging Spectroradiometer (MODIS) sensors were launched to sun-synchronous orbits on the Terra (10:30 local standard time) in 1999 and on the Aqua (13:30 local standard time) in 2002. A 2330 km viewing swath provides near-global coverage in 1–2 days. Spatial resolutions vary with bands (from 250 m to 1 km at nadir) and become larger at the edge of the swath, by a factor of ∼2 along-track and ∼5 across-track. Three algorithms are applied to retrieve 550 nm AOD: the Deep Blue (DB) and Dark Target (DT) algorithms over land, and the DT over-water algorithm^22,23^. MODIS retrieves AOD with an estimated uncertainty of ±0.05 ± 0.20 × AOD over the land^22^. The collection 6.0 AOD datasets are available from https://ladsweb.modaps.eosdis.nasa.gov at a nominal (nadir) spatial resolution of 10 × 10 km. We created gridded AOD covering Beijing (115.2°E-117.6°E, 39.4°N-41.2°N) with a spatial resolution of 0.1° × 0.1° by using 13-year Level 2 Aqua merged DT and DB AOD at 550 nm (2002–2014). Mean AQUA AOD exhibits a strong spatial gradient, with the highest values over the southeast urban districts (Fig. 8).

### Aeronet Aod

AERONET is a ground-based internationally federated, globally distributed network of sun photometers. AERONET AOD is derived from direct beam solar measurements at wavelengths from ultraviolet to infrared^24^. We used the cloud-screened and quality checked level 2.0 AOD product (http://aeronet.gsfc.nasa.gov/)^25^. Instantaneous AOD at 550 nm at Beijing during 2002–2014 was interpolated from AOD at 440 nm and at 675 nm. AOD products at SDZ and Xianghe (during 2005–2011) were served as the validation datasets for the data fusion (Table 2). Statistics of AODs at Beijing (2002–2014), Xianghe and SDZ (2005–2011) are presented in Table 2. SDZ is one of Chinese Aerosol Research Science Network (CARSNET) stations. The CARSNET uses the same sunphotometer and algorithm as AERONET to retrieve AOD with the comparable accuracy to that of AERONET^26,27^.

**Table 2:** Site information of Beijing, Xianghe and SDZ.

	Lon	Lat	Year	N_A_	N_M_	N_F_	Purpose
Beijing	116.38	39.97	2002–2014	2977	2397	3367	Model development
SDZ	117.12	40.65	2005–2011	1866	1277	1735	Validation
Xianghe	116.96	39.75	2005–2011	1840	1262	1894	Validation

(Noted: N_A_, N_M_, N_F_ represent AOD sampling by AERONET, MODIS and AERONET-MODIS fusion, respectively).

### Data fusion approach

We establish a linear formula (slope and intercept) on the basis of daily AOD data pairs of AERONET AOD at Beijing site and MODIS gridded AOD within the Beijing Area (115.2°E-117.6°E, 39.4°N-41.2°N) during 2002–2014 at each grid. The analysis is performed based on daily paired AODs in four seasons, i.e., spring (March-April-March); summer (June-July-August); autumn (September-October-November) and winter (December-January-February). Pearson coefficient maps are derived from linear correlation analysis between two variables above (Fig. 2). A threshold value of correlation coefficient (R ≥ 0.5) is set to determine whether AERONET AOD can be used in the estimation of regional PM_2.5_. For grids with R ≥ 0.5, we use the linear-fit AOD values based on AERONET Beijing to fill missing values of MODIS AOD retrievals.

### The mixed effects Model

A mixed effects model to investigate the AOD-PM_2.5_ relationship is as follows.

1 $$\begin{array}{c}{{\rm{PM}}}_{{\rm{i}},{\rm{j}}}=({\rm{\alpha }}+{{\rm{u}}}_{{\rm{j}}})+({\rm{\beta }}+{{\rm{v}}}_{{\rm{j}}}){{\rm{AOD}}}_{{\rm{i}},{\rm{j}}}+{{\rm{s}}}_{{\rm{i}}}+{{\rm{\varepsilon }}}_{{\rm{i}},{\rm{j}}}\\ ({{\rm{u}}}_{{\rm{j}}}{{\rm{v}}}_{{\rm{j}}} \sim {\rm{N}}[(00),\sum ])\end{array}$$

where PM_i,j_ represents PM_2.5_ value at site i on day j; α and β represent fixed intercept and slope respectively; u_j_ and v_j_ are the random intercept and slope. s_i_ ~ N (0, ${{\rm{\sigma }}}_{{\rm{s}}}^{2}$) and ε_i,j_ ~ N (0, σ^2^) represent the random intercept of site i and the error term at site i on day j. ${{\rm{\sigma }}}_{{\rm{s}}}^{2}$ and σ^2^ denote the variances for s_i_ and ε_i,j_. ∑ is the variance-covariance matrix for the day-specific random effects^7^. We select the site-specific satellite AOD values for each surface site where it falls within a 10 × 10 km^2^ grid to collocate PM_2.5_ concentrations. If there are more than one site within a single 10 × 10 km^2^ grid, the PM_2.5_ values of those sites are averaged. With this process, there remain 25 pairs of AOD and PM_2.5_ data for the model development.

### Data availability

The datasets generated during and/or analyzed in the current study are available from the corresponding author on reasonable request.
